# Patterns of cervical lymph node metastasis in supraglottic laryngeal cancer and therapeutic implications of surgical staging of the neck

**DOI:** 10.1007/s00405-021-06753-1

**Published:** 2021-03-27

**Authors:** Cornelius H. L. Kürten, Eleni Zioga, Thomas Gauler, Martin Stuschke, Maja Guberina, Johannes M. Ludwig, Eric Deuss, Stefan Mattheis, Stephan Lang, Timon Hussain

**Affiliations:** 1 Department of Otorhinolaryngology, Head and Neck Surgery, University Hospital Essen, University of Duisburg-Essen, Hufelandstraße 55, 45147 Essen, Germany; 2 Department of Radiation Oncology, University Hospital Essen, University of Duisburg-Essen, Essen, Germany; 3 Department of Radiology, University Hospital Essen, University of Duisburg-Essen, Essen, Germany

**Keywords:** Head and neck cancer, Laryngeal cancer, Neck dissection, Transoral robotic surgery, Staging

## Abstract

**Purpose:**

Accurate therapeutic management of the neck is a challenge in patients with supraglottic laryngeal cancer. Nodal metastasis is common at all disease stages, and treatment planning relies on clinical staging of the neck, for both surgical and non-surgical treatment. Here, we compared clinical and surgical staging results in supraglottic carcinoma patients treated with primary surgery to assess the accuracy of pre-therapeutic clinical staging and guide future treatment decisions.

**Methods:**

Retrospective analysis of clinical, pathological, and oncologic outcome data of 70 patients treated with primary surgery and bilateral neck dissection for supraglottic laryngeal cancer. Patients where clinical and pathological neck staging results differed, were identified and analyzed in detail.

**Results:**

On pathologic assessment, patients with early stage (pT1/2) primaries showed cervical lymph node metastases in 55% (*n* = 17/31) of cases, compared to 67% (*n* = 26/39) of patients with pT3/4 tumors. In 24% (*n* = 17/70) of all patients, cN status differed from pN status, resulting in an upstaging in 16% of cases (*n* = 11/70) and a downstaging in 9% (*n* = 6/70) of cases. 14% of patients with cN0 status had occult metastases (*n* = 5/30). As assessed by a retrospective tumor board, in case of a non-surgical treatment approach, the inaccurate clinical staging of the neck would have led to an over- or undertreatment of the neck in 20% (*n* = 14/70) of all patients.

**Conclusion:**

Our data re-emphasize the high cervical metastasis rates of supraglottic laryngeal cancer across all stages. Inaccurate clinical staging of the neck is common and should be taken into consideration when planning treatment.

## Introduction

Due to the extensive regional lymphatic network, supraglottic laryngeal carcinomas have a tendency to develop neck metastases at all disease stages, hereby negatively impacting patients’ prognosis and influencing treatment decisions [1, 2]. For patients treated with a primary surgical approach, nodal metastasis is routinely managed by neck dissection, oftentimes followed by adjuvant radiotherapy. While ipsilateral elective neck dissection is generally recommended as part of a primary surgical approach for supraglottic carcinomas by current guidelines, the question whether bilateral neck dissection of the clinically N0 neck is warranted has been debated controversially [3–5]. For head and neck cancer in general, based on decision analysis models [6], a 20% chance of occult metastasis has been proposed to be a justifiable threshold for elective neck dissection. Given the significant changes in neck dissection technique in the past decades [7], today, even occult metastasis rates lower than 20% have been deemed appropriate to justify elective neck dissection of the respective levels at risk [5, 8]. This changed risk–benefit analysis is mainly driven by a more selective and functionally orientated neck dissection classification and surgical technique as well as improved imaging techniques [8–10].

At our institution, in anticipation of high rates of occult nodal metastasis, all patients undergoing primary surgery for supraglottic laryngeal carcinomas also routinely underwent bilateral neck dissection. Based hereon, we compared the pre-operative clinical neck staging (cN) results, as assessed by ultrasound, and CT scans, to post-operative neck staging results, as assessed by pathology (surgical staging, pN), hereby determining the accuracy of clinical staging of the neck and the rate of occult metastasis in our patient collective. Patients undergoing a primary surgical approach including bilateral neck dissection have the benefit of a definitive pathological nodal status after surgery which guides adjuvant treatment planning, [11] i.e., the addition of radiotherapy if nodal involvement is shown [12], and radiochemotherapy with the inclusion of cisplatin if extracapsular spread is established, or the resection is incomplete [13]. On the other hand, in patients undergoing a primary non-surgical treatment approach, clinical nodal staging determines the entire therapeutic regimen and is, therefore, of particular interest. Potential discrepancy rates between clinical and pathological staging results must be kept in mind during therapy planning to avoid over/undertreatment.

## Materials and methods

### Patients

We selected patients who underwent primary surgery for supraglottic laryngeal cancer at our institution between 2004 and 2019. Inclusion criteria were complete staging and initial treatment at our cancer center, no secondary malignancy at the time of diagnosis, availability of clinical as well as pathological staging data and the treatment recommendation of our interdisciplinary tumor board to perform primary tumor resection and bilateral neck dissection with curative intent. Routine staging included contrast-enhanced CT scans of the head and neck, contrast enhanced CT scan of the thorax to rule out lung metastasis or second primaries, ultrasound of the abdomen to rule out abdominal metastasis or second primaries and ultrasound of the neck. All clinical and pathological parameters were recorded by chart review. Staging was determined according to the current staging system at time of diagnosis (AJCC Staging Manual 6th Edition up to 2009, AJCC Staging Manual 7th Edition up to 2017, AJCC Staging Manual 8th Edition starting up to 2019).

### Retrospective comparison of clinical and pathological neck staging

An experienced radiologist and an experienced radiation oncologist blinded to pN-status re-assessed the clinical node status of patients with divergent cN vs. pN. In 3/17 cases, the head and neck CT scan was no longer available in the electronic radiology database and we therefore, relied on the written report at the time of diagnosis. We conducted a virtual retrospective tumor board to determine the impact of the postoperative pN-status on hypothetical treatment decision had the patient received primary radiotherapy.

### Statistical analysis and data visualization

Survival analysis was performed using GraphPad Prism 8 for Windows (Version 8.4.3, GraphPad Software, San Diego, CA, USA). 67 patients, for which survival data were available, were included. The sankey diagram was created using SankeyMATIC (http://sankeymatic.com). Figure 2, for easier visualization, shows the stages c/pN3a and c/p3Nb in a combined fashion as c/pN3.

## Results

### Patients and treatment

70 patients who underwent surgery with curative intent for supraglottic laryngeal cancer between 2004 and 2019 met the inclusion criteria. 73% (*n* = 51) of patients were male 27% (*n* = 19) were female. Age distribution was as follows: ≤ 49 years: 3% (*n* = 2); 50–59 years: 21% (*n* = 15), 60–69 years: 49% (*n* = 34), 70–79 years: 23% (*n* = 16) and ≥ 80 years: 4% (*n* = 3). 50% (*n* = 35) of patients presented with clinical stage T1 and T2 primary tumors and 50% (*n* = 35) of patients presented with stage T3 and T4 primary tumors at the time of diagnosis. Clinically assessed nodal status was cN0 for 43% (*n* = 30) of patients, cN1 for 6% (*n* = 4), cN2 for 46% (*n* = 32) of patients and cN3 for 6% (*n* = 4) of patients. According to AJCC (American Joint Committee on Cancer) criteria, 4% (*n* = 3) of patients were allocated to stage I, 17% (*n* = 12) to stage II, 21% (*n* = 15) to stage III and 57% (*n* = 40) to stage IV. 47% (*n* = 33) of patients underwent total laryngectomy, while 53% (*n* = 37) of patients underwent transoral tumor resection (21 patients with TORS, 16 patients with TLM). On post-operative pathology, 55% (*n* = 17/31) of patients with early-stage primary cancers (i.e., pT1/2), had positive lymph nodes after surgical staging, compared to 67% (*n* = 26/39) of patients with advanced stage primary cancers, i.e. pT3/4 cancers. Patient and disease characteristics are provided in Table 1. All patients underwent bilateral neck dissection. One patient had resectable distant metastatic disease and received a lobectomy for lung metastasis. The postoperative tumor board recommended adjuvant therapy in 71% (*n* = 50) of cases.

**Table 1:** Clinical patient characteristics

Patient and disease characteristics	
Gender	
Male	73%
Female	27%
Age (avg. in years)	65
Clinical T stage	
cT1	10%
cT2	40%
cT3	34%
cT4	16%
Clinical N stage	
cN0	43%
cN1	6%
cN2	46%
cN3	6%
AJCC stage	
I	4%
II	17%
III	21%
IVa	49%
IVb	7%
IVc	1%
Surgical treatment	
Total laryngectomy	47%
TORS	30%
TLM	23%
Adjuvant therapy	71%

*AJCC* American Joint Committee on Cancer, *TORS* transoral robotic surgery, *TLM* transoral laser microsurgery

### Survival analysis

**Fig. 1:**
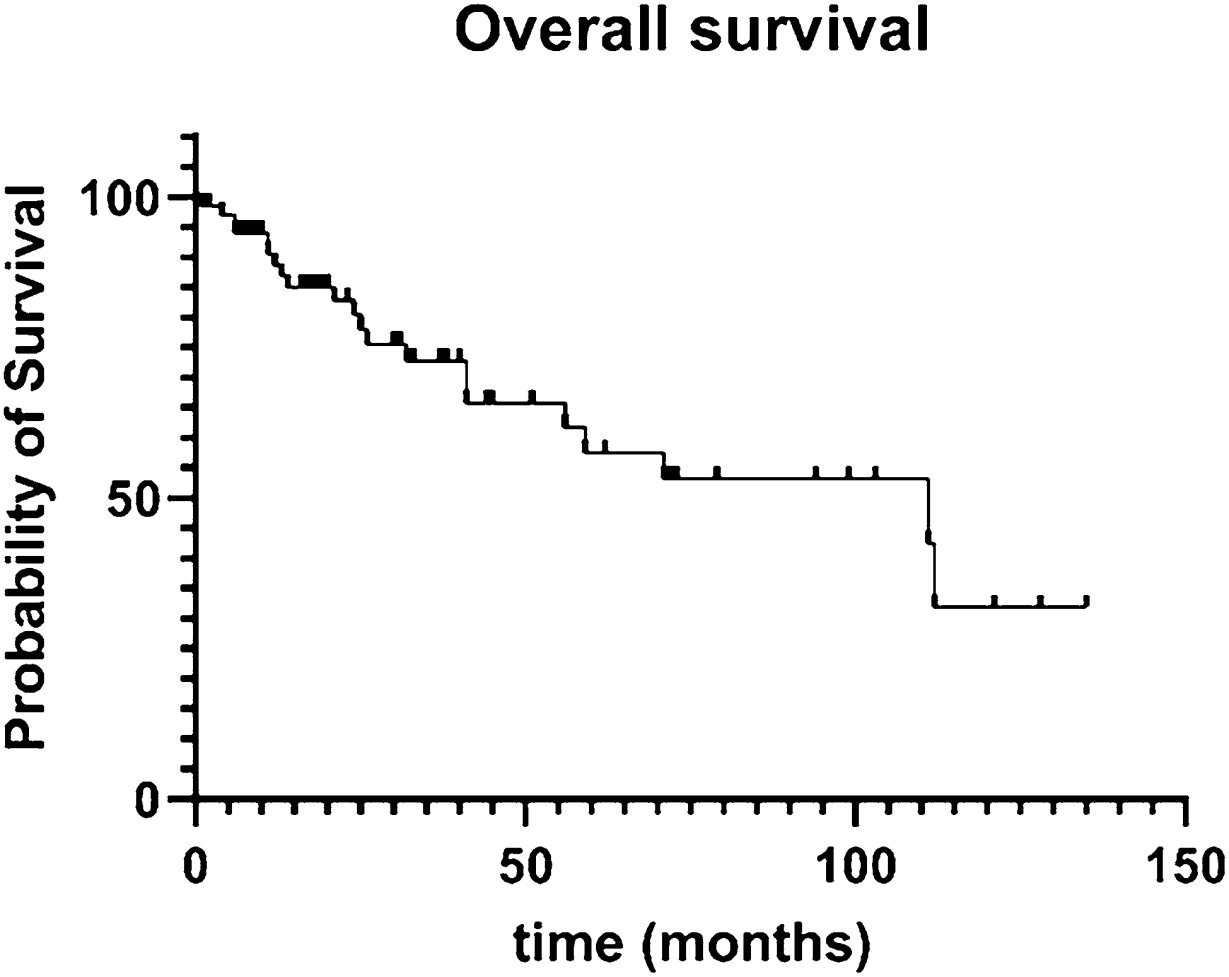
Kaplan–Meier curve showing overall survival data for 67 patients. At the 24-month cut-off, 80% of patients were alive, at the 60-month cut-off, 58% of patients were alive, at 135 months, 32% of patients were alive

A survival analysis was performed (Fig. 1), showing 80%, 58%, and 30% of patients to be alive after 24, 60, and 135 months of follow-up, respectively.

### Clinical vs. pathological nodal staging

**Fig. 2:**
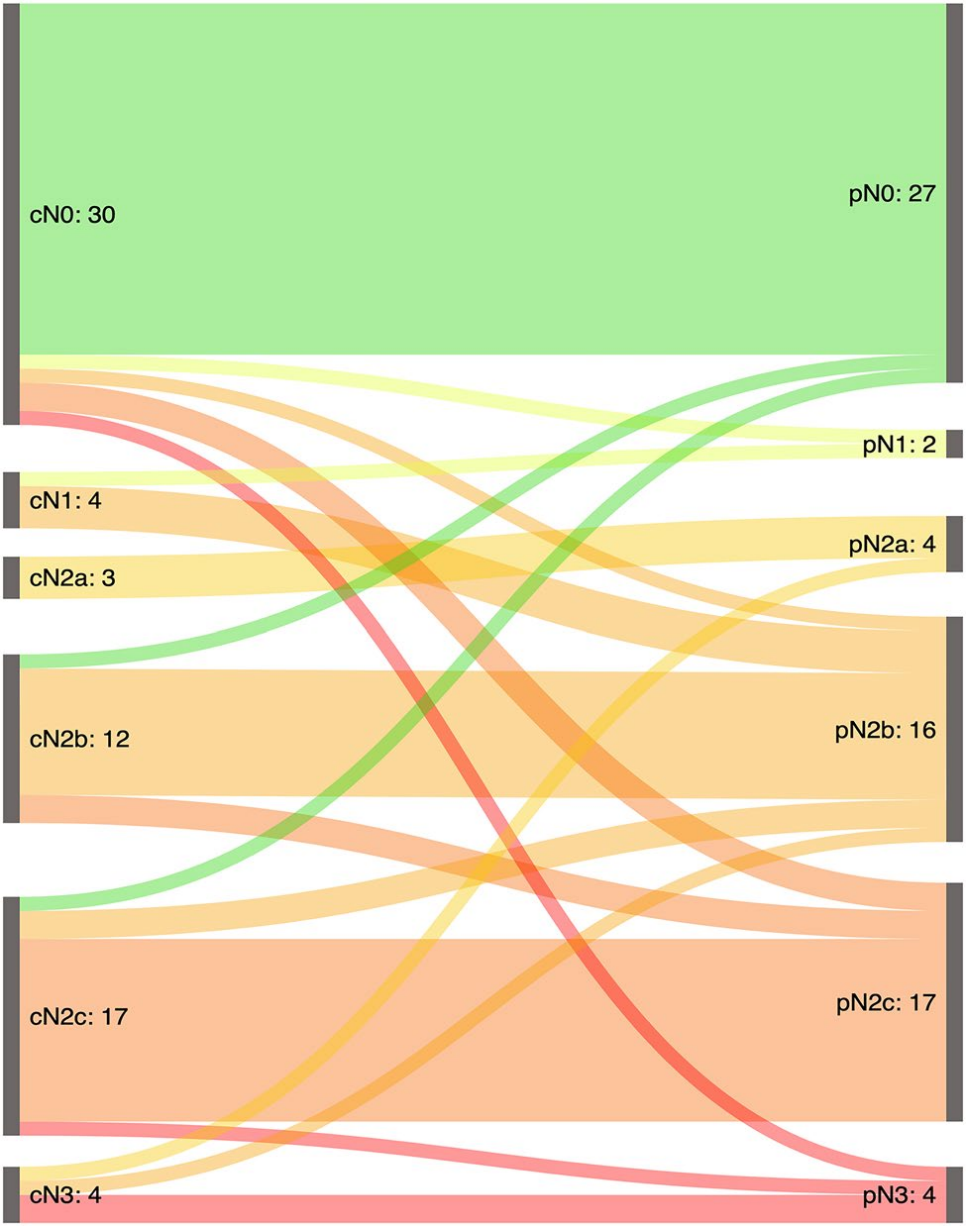
Sankey diagram graphically depicting the changes from clinical to pathological staging for all patients. Overall, cN stage differed fom pN stage in 24% (*n* = 17/70) of cases. Upstaging occurred in 16% of cases (*n* = 11/70) and was more common than downstaging, which occurred in 9% (*n* = 6/70) of cases

Clinical and pathological nodal status differed in 24% (*n* = 17/70) of all cases: Upstaging, i.e., pN > cN occurred in 16% of cases (*n* = 11/70) and was more common than downstaging, i.e., pN < cN which occurred in 9% (*n* = 6/70) of cases (Table 2). Figure 2 visualizes the re-staging occurring between clinical and pathological nodal status. One can appreciate that in our patient collective, the occurrence of single metastases (smaller or larger than 3 cm) are less common than multiple metastases in the individual patient. Of note, pathological nodal stage N2b, i.e., multiple ipsilateral metastatic lymph nodes seems to be most difficult to assess clinically, as three patients were wrongly classified as cN2b while seven patients with pN2b were previously classified differently on clinical staging, resulting in a consistency rate of 56% when comparing clinical to pathological staging.

**Table 2:** Detailed patient characteristics for patients staged cN0 (*n* = 5) where pathological staging revealed metastatic lymph nodes

Patient	Age	Gender	Location	Tumor lateralization	cT	pT	cN	pN	LN side	LN details
Pt1	64	F	Supraglottis	Lateralized left	2	1	0	1	left	Level 1 (1 LN, micrometastasis < 1 mm)
Pt2	77	M	Supraglottis	Not lateralized	3	3	0	2b	left	Level 1 (2 LN, metastases both < 10 mm)
Pt3	70	M	Supraglottis	Not lateralized	4a	4a	0	2c	bilateral	Level IIa right (1 LN, cystic metastasis), Level IIa left (1 LN, 8 mm)
Pt4	64	F	Supraglottis	Not lateralized	2	3	0	2c	bilateral	Level II left (1 LN 16 mm) Level III left (1 LN) 14 mm, Level III right (1 LN metastasis)
Pt5	68	M	Supraglottis	Not lateralized	4	4a	0	3b	left	Level II (2 metastases), Level III (2 metastases, 1 ENE +)

*F* female, *M* male, *LN* lymph node, *ENE* extra nodal extension

Changes from cN0 status to pN + -status are clinically the most relevant and were, therefore, analyzed in detail. 14% of all patients clinically staged N0 (*n* = 5/30) showed metastatic nodes on final pathology and details for these are summarized in Table 2. Remarkably, all metastatic nodes detected in this patient collective were less than 1.6 cm in diameter. In one case, upstaging from N0 to N3 was due to pathological extracapsular spread in one node.

### Therapeutic implications of neck staging differences

Therapeutic implications for all patients where final pathology findings differed from the initial clinical neck staging results (24% of all patients, *n* = 17/70) are listed in Table 3. All patients in our analysis were treated with primary surgery including bilateral neck dissection and in 76% (*n* = 13/17) of patients where pN differed from cN, adjuvant treatment was affected the altered neck staging results. We also performed a virtual retrospective tumor board to assess whether the therapeutic regime would have been altered if patients had been treated with primary a primary non-surgical approach and the true nodal status had been known: here, treatment alterations would have occurred in 82% (*n* = 14/17) of patients. Notably, 11% (*n* = 8/70) of all patients would have received more intensive treatment according to current guidelines based on their pN status compared to their cN status.

**Table 3:** Summary of therapeutic relevance of c/pN divergence and resulting changes of adjuvant and primary radiation therapy

cN	→ pN	No. of patients	Up/downstaging	Effect of cN → pN change on adjuvant treatment	Hypothetical effect of cN → pN change on primary radiotherapy treatment
cN0	pN1	1	Up	Higher radiation dose ipsilateral side	Higher radiation dose
	pN2b	1	Up	Higher radiation dose ipsilateral side	Higher radiation dose
	pN2c	2	Up	Higher radiation dose	Higher radiation dose
	pN3b	1	Up	Higher radiation dose, additional Chemotherapy	Higher radiation dose, additional Chemotherapy
cN1	pN2a	3	Up	None	None
cN2b	pN0	1	Down	Lower radiation dose ipsilateral side	Lower radiation dose
	pN2c	2	Up	Higher radiation dose contralateral side	Higher radiation dose contralateral side
cN2c	pN0	1	Down	Lower radiation dose	Lower radiation dose
	pN2b	2	Down	Lower radiation dose	Lower radiation dose contralateral side
	pN3b	1	Up	Higher radiation dose	Higher radiation dose, additional Chemotherapy
cN3	pN2a	1	Down	Lower radiation dose	Lower radiation dose
	pN2b	1	Down	None	Lower radiation dose

Hypothetical effects in case of a primary radiotherapy treatment approach were assessed via a retrospective virtual tumor board

## Discussion

The findings of our retrospective study confirm the pronounced tendency of supraglottic laryngeal cancer to develop regional metastases at all stages. In our patient collective, patients with early stage primary tumors, i.e, stages T1 and T2, developed lymph node metastases in 55% of cases at the time of surgery, compared to 67% of patients with advanced local tumors, i.e., T stages 3 and 4. This is in line with findings by others, who showed similarly high regional metastasis rates [14]. While neck dissection is indisputably part of any primary surgical treatment approach for supraglottic laryngeal cancer and part of current treatment guidelines, unilateral neck dissection for early stage primary tumors with a clinical N0 neck has been advocated [15], since occult nodal metastases are most likely to occur ipsilaterally in patients with T1 and T2 primary tumors. In our study, 17% (*n* = 5/30) of patients across all stages who were clinically staged N0 had positive lymph nodes on final pathological assessment, i.e., occult nodal metastases. These numbers are comparable, or slightly lower than those reported by other authors, who found occult metastases in up to 30% of patients with clinical N0 status at the time of surgery [16–18]. Our data suggest a positive correlation between the rate of occult nodal metastasis and the size of the primary tumor: Four of the five patients with occult nodal metastasis had advanced stage local disease. The one patient with an early-stage primary tumor had an occult nodal micro-metastasis. Notably, all occult metastases were located ipsilaterally. The single case of occult nodal disease within the group of patients with smaller primary tumors is most likely attributable to the sample size of our patient collective; Zhang et al. who focused on a larger cohort of cN0 supraglottic tumors revealed occult nodal metastasis rates of 13.0% and 20.5% for pT1 and pT2 tumors, respectively [18]. Here too, though, contralateral metastases and contralateral regional recurrence were rare in patients with clearly lateralized, early-stage primary tumors. Importantly, this does not apply to patients where the primary tumor is located close to, or even at the anatomical midline, as has been pointed out before [19]. However, if these anatomical and oncologic criteria are met, i.e., a clearly lateralized early stage primary tumor and clinical N0 status, unilateral neck dissection seems to be justifiable. In all other cases, i.e., primary tumors close to the midline, or advanced stage primary tumors, or clinically positive nodal status, bilateral neck dissection should be performed.

Compared to a non-surgical treatment approach, primary surgery for supraglottic laryngeal cancer has the inherent advantage of providing definitive pathological staging of the neck and the aim of this study was to assess the differences between cN and pN stage and analyze the impact of surgical staging of the neck on the therapeutic regimen. In our patient collective, pre-therapeutic clinical N-status and post-surgical N-status as assessed by pathology differed in 24% of cases. These data are in line with analyses by others for supraglottic laryngeal carcinomas [16]; similar discrepancy rates have also been reported for oropharyngeal cancer patients who underwent primary surgery [20]. Importantly, in 16% of all patients, pN stage exceeded cN stage, i.e., surgery revealed additional neck metastases which elevated the patients’ disease stage, according to AJCC guidelines. Notably, staging discrepancies affected treatment: for the majority of upstaged patients, the dose of adjuvant radiation to the neck was higher than it would have been according to clinical N-stage. Similarly, in patients where pN stage was lower than cN stage, i.e., 9% of all patients, the adjuvant therapeutic regimen was also affected, and treatment intensity was reduced based on pN stage. Of course, for our patients, these deliberations are purely hypothetical, since all patients underwent a primary surgical approach and neck dissection was routinely performed, guaranteeing a definitive pathological neck staging result. However, these findings do serve as an affirmation of the value of surgical staging of the neck, particularly in an era, where more organ-preserving surgical approaches are available for supraglottic laryngeal cancer patients as an alternative to primary radiation. The advancements in transoral surgery, particularly promoted by the recent developments in TORS for supraglottic laryngeal cancer, have provided a surgical, yet organ-preserving primary treatment approach for selected patients who may have otherwise be treated with primary radiation therapy. In our patient cohort, 53% of patients underwent transoral surgery, the majority via TORS with the Medrobotics^®^ FLEX system, and 21% of those patients had stage T3 primary tumors, which could be successfully resected via the transoral approach, emphasizing the comprehensiveness of the technology, as has been previously shown [21–23]. For these patients in particular, transoral surgery combined with bilateral neck dissection provided an effective primary treatment while allowing for highly accurate adjuvant therapy based on the results of pathologic staging of the neck.

## Conclusion

Our data confirm the notion that supraglottic laryngeal cancers show a pronounced tendency to spread to regional lymph nodes of the neck at every disease stage. Therefore, in case of a primary surgical treatment approach, bilateral neck dissection is warranted with the exception of early stage, clearly lateralized tumors staged cN0; here, ipsilateral neck dissection may be considered. Our analysis revealed a considerable discrepancy between clinical and pathological neck staging results which affected the adjuvant treatment regimen in our patients. Notably, as determined via a retrospective tumor board, if a primary non-surgical treatment approach had been chosen for our patients, nearly 20% of patients would have received an under/overtreatment of the neck because cN-staging was inaccurate. These findings should be taken into consideration when establishing the primary treatment approach for patients with supraglottic laryngeal cancer, particularly for patients where both surgical and non-surgical treatment approaches are available.
